# Integrated proteomic analysis reveals interactions between phosphorylation and ubiquitination in rose response to *Botrytis* infection

**DOI:** 10.1093/hr/uhad238

**Published:** 2023-11-14

**Authors:** Rui Li, Juanni Yao, Yue Ming, Jia Guo, Jingjing Deng, Daofeng Liu, Zhengguo Li, Yulin Cheng

**Affiliations:** 1 Key Laboratory of Plant Hormones and Development Regulation of Chongqing, School of Life Sciences, Chongqing University, Chongqing 401331, China; 2 Chongqing Engineering Research Center for Floriculture, Key Laboratory of Horticulture Science for Southern Mountainous Regions of Ministry of Education, College of Horticulture and Landscape Architecture, Southwest University, Chongqing 400715, China

## Abstract

As two of the most abundant post-translational modifications, phosphorylation and ubiquitination play a significant role in modulating plant–pathogen interactions and increasing evidence indicates their crosstalk in plant immunity. Rose (*Rosa* sp.) is one of the most important ornamental plants and can be seriously infected by *Botrytis cinerea*. Here, integrated proteomics analysis was performed to detect global proteome, phosphorylation, and ubiquitination changes in rose upon *B. cinerea* infection and investigate the possible phosphorylation and ubiquitination crosstalk. A total of 6165 proteins, 11 774 phosphorylation and 10 582 ubiquitination sites, and 77 phosphorylation and 13 ubiquitination motifs were identified. *Botrytis cinerea* infection resulted in 169 up-regulated and 122 down-regulated proteins, 291 up-regulated and 404 down-regulated phosphorylation sites, and 250 up-regulated and 634 down-regulated ubiquitination sites. There were 12 up-regulated PR10 proteins and half of them also showed reduced ubiquitination. A lot of kinases probably involved in plant pattern-triggered immunity signaling were up-regulated phosphoproteins. Noticeably, numerous kinases and ubiquitination-related proteins also showed a significant change in ubiquitination and phosphorylation, respectively. A cross-comparison of phosphoproteome and ubiquitylome indicated that both of two post-translational modifications of 104 proteins were dynamically regulated, and many putative pattern-triggered immunity signaling components in the plant plasma membrane were co-regulated. Moreover, five selected proteins, including four PR10 proteins and a plasma membrane aquaporin, were proven to be involved in rose resistance to *B. cinerea*. Our study provides insights into the molecular mechanisms underlying rose resistance to *B. cinerea* and also increases the database of phosphorylation and ubiquitination sites in plants.

## Introduction

Protein post-translational modifications (PTMs) play a significant role in cellular and physiological processes mainly by influencing the activity, concentration, and subcellular localization of the protein and its interaction with partners [1]. Phosphorylation and ubiquitination are two of the most abundant and well-studied PTMs in eukaryotes and contribute to cellular signaling and protein degradation, respectively [2]. Both phosphorylation and ubiquitination are reversible. Phosphorylation is governed by protein kinases and reverse phosphatases, and ubiquitination is governed by three main types of enzymes: ubiquitin-activating enzymes (E1), ubiquitin-conjugating enzymes (E2), and ubiquitin ligases (E3), and reverse deubiquitinases [3, 4]. Plant diseases caused by pathogenic microbes cause severe crop loss worldwide and result in huge economic losses annually, and both phosphorylation and ubiquitination play an important role in plant resistance to pathogen infection [1, 5]. Receptor-like kinases (RLKs), of which many are plant cell surface-localized pattern recognition receptors (PRRs) and co-receptors in pattern-triggered immunity (PTI), receptor-like cytoplasmic kinases (RLCKs), and the downstream mitogen-activated protein kinase (MAPK) cascades, are important components of plant PTI signaling [5, 6]. Reversible phosphorylation of other plant immune-related proteins, such as the central immune component RIN4, nicotinamide adenine dinucleotide phosphate (NADPH) oxidase RBOHD, transcription factors WRKY33 and IPA1, plant-specific Ga protein XLG2, and phytochrome-interacting factor PIF3, are also required for plant disease resistance [7–12]. In addition, a lot of E3 ubiquitin ligases, including U-box type, RING type, and CRL type E3 ligases, were shown to be involved in many aspects of plant immunity mainly by regulating the degradation or subcellular localization of their substrate proteins [13]. Meanwhile, several E2 ubiquitination enzymes and the regulatory particle non-ATPase (RPN) subunit of the 26S proteasome were also proven to regulate plant immunity [14–16].

In addition to a single regulatory PTM, multiple PTMs can modify the same protein in an orchestrated manner, which is termed ‘PTM crosstalk’ and usually functions as a fine-tuning strategy in many cellular and physiological processes [4]. Although the understanding of PTM crosstalk in plants is still at the initial phase, there is increasing evidence for crosstalk between phosphorylation and ubiquitination in plant immunity [1]. Previous studies have shown that phosphorylation and ubiquitination mainly converge on plant PTI signaling [1]. Moreover, crosstalk between protein kinases and E3 ubiquitin ligases in plant immunity has been identified in several model plant species [1]. E3 ubiquitin ligase-mediated ubiquitination of RLKs/RLCKs causes their degradation or changes their subcellular localization, and RLK/RLCK-mediated phosphorylation of E3 ubiquitin ligases affects their patterns of interaction with substrates or enzymatic activity [1]. Moreover, a recent study showed that *Arabidopsis* NADPH oxidase RBOHD was regulated by both phosphorylation and ubiquitination and their crosstalk fine-tuned the production of reactive oxygen species (ROS) in plant immunity [17]. Thus, identification of co-regulated proteins by both phosphorylation and ubiquitination during the plant response to pathogen infection will lead to better understanding of PTM crosstalk in plant immunity.

*Botrytis cinerea* is a typical necrotrophic fungus and is considered to be the second most important fungal plant pathogen; it can cause gray mold disease on >1000 plant species [18, 19]. Current understanding of mechanisms of plant immunity to *B. cinerea* mainly comes from the model plant *Arabidopsis thaliana* and tomato, and PTI is the key component of plant immune responses to *B. cinerea* infection [20]. Notably, some protein kinases, including RLKs, RLCKs, and MAPK cascades [20, 21] and several RING type and U-box type E3 ubiquitin ligases [22, 23], were proven to be important regulators of plant resistance to *B. cinerea*, highlighting the contribution of phosphorylation and ubiquitination to plant resistance to *B. cinerea*. Rose (*Rosa* sp.) is an important ornamental crop worldwide, with great economic, cultural, and symbolic value [24], and rose petals can be seriously infected by *B. cinerea*, especially during the post-harvest stage [25]. Although WRKY transcription factors and phytohormones, including jasmonic acid, ethylene, abscisic acid, brassinosteroid, and cytokinin, were shown to regulate the resistance to *B. cinerea* in rose petals [26–29], the molecular mechanisms of rose disease resistance remain relatively unexplored.

With the rapid development of proteomics techniques, the phosphoproteome and ubiquitylome enable a systematic investigation of phosphorylation and ubiquitination under specific physiological conditions in plants, including the plant’s response to pathogen infection [9, 15, 30]. In this study, integrated proteomic analysis was performed to detect global proteome, phosphorylation, and ubiquitination changes in rose petals upon *B. cinerea* infection and investigate the possible phosphorylation and ubiquitination crosstalk. A total of 6165 proteins, 11 774 phosphorylation and 10 582 ubiquitination sites, and 77 phosphorylation and 13 ubiquitination motifs were identified, which increases the database of phosphorylation and ubiquitination sites in plants. Our results demonstrated close interactions between phosphorylation and ubiquitination in the rose response to *B. cinerea* infection, and co-regulation of many putative PTI signaling components in the plant plasma membrane by both phosphorylation and ubiquitination was observed. Moreover, five selected proteins, including four pathogenesis-related (PR) PR10 proteins and a plasma membrane aquaporin, were shown to be involved in rose resistance to *B. cinerea*. This work noticeably advances our understanding of the mechanisms underlying rose resistance to *B. cinerea*.

## Results

### Experimental strategy

**Figure 1:**
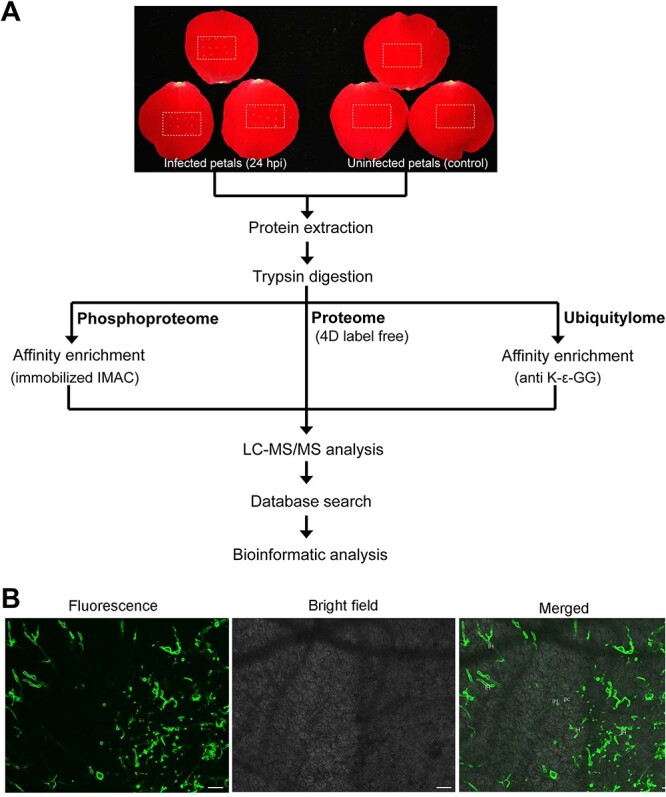
Overview of integrated proteomic analysis in this study. **A** Workflow of proteome, phosphoproteome, and ubiquitylome analyses. Dotted boxes indicate specific sampling locations. **B** Cytological observation of *B. cinerea* growth in inoculated rose petals at 24 hpi. IH, infection hyphae; pc, parenchyma cell. Scale bar = 50 μm.

Because PTI, the first line of plant disease defense, is considered the key component of plant immune responses to *B. cinerea* infection [20], uninfected rose petals (control) and infected rose petals at 24 h post-inoculation (24 hpi) with *B. cinerea*, which is regarded as the time of the early response to *B. cinerea* infection, were sampled for proteome, phosphoproteome, and ubiquitylome analyses (Fig. 1A). At 24 hpi, only slight disease lesions began to be observed on rose petals (Fig. 1A) and *in planta* visualization of fungal growth confirmed the infection of *B. cinerea* in rose petals (Fig. 1B). 4D label-free quantitative proteomics, which is one of the latest proteomic techniques [31], was used for quantification of proteome changes in this study. Immobilized metal ion affinity chromatography (IMAC) [32] and anti-diglycine remnant (anti-K-ε-GG) antibody [33] were used for enrichment of phosphorylated and ubiquitinated peptides in the phosphoproteome and ubiquitylome, respectively. Phosphorylation and ubiquitination quantifications in phosphoproteome and ubiquitylome were normalized by protein quantifications to eliminate the interference caused by protein abundance differences in the proteome.

### Changes in proteome profile of rose response to *B. cinerea* infection

**Figure 2:**
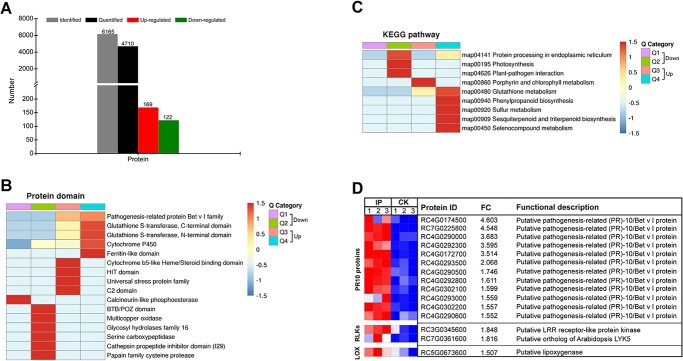
*B. cinerea* infection-regulated rose proteome. **A** Characterization of rose proteins in the proteome. **B** Protein domain enrichment analysis of DEPs. **C** KEGG enrichment analysis of DEPs. **D** Heat map showing the expression of some DEPs in infected petals (IP) and uninfected petals (CK). Q1, Q2, Q3, and Q4 in **B** and **C** indicate FC < 0.5, 0.5 ≤ FC < 0.667, 2 ≥ FC > 1.5, and FC > 2, respectively.

Quantitative proteomics analysis of uninfected and infected rose petals identified a total of 6165 rose proteins (Supplementary Data Table S1), of which 4710 can be quantified (Fig. 2A). Based on the criteria of fold change (FC) >1.5 and *P* value <0.05, 291 differentially expressed proteins (DEPs), including 169 up-regulated and 122 down-regulated proteins, were identified in the rose response to *B. cinerea* infection (Fig. 2A; Supplementary Data Table S2). For enrichment analysis, DEPs were divided into four clusters (Q1–Q4) based on the fold change, and Q1, Q2, Q3, and Q4 indicate FC < 0.5, 0.5 ≤ FC < 0.667, 2 ≥ FC > 1.5, and FC > 2, respectively (Fig. 2B and C). Protein domain enrichment analysis of rose DEPs in the proteome showed that PR-10/Bet_v_1 proteins and two large enzymatic protein families, including glutathione *S*-transferase and cytochrome P450, which play important roles in plant disease resistance [34, 35], were noticeably up-regulated in rose upon *B. cinerea* infection (Fig. 2B). Kyoto Encyclopedia of Genes and Genomes (KEGG) enrichment analysis showed that up-regulated DEPs were mainly enriched in the ‘glutathione metabolism, ‘phenylpropanoid biosynthesis’, ‘sulfur metabolism’, ‘sesquiterpenoid and triterpenoid biosynthesis’, and ‘selenocompound metabolism’ pathways (Fig. 2C). The induction of PR proteins is an important component of plant defense responses [36], and further analysis showed that a total of 12 rose PR10 proteins were uniformly up-regulated upon *B. cinerea* infection (Fig. 2D). In addition, two RLKs probably involved in plant PTI signaling, of which one is the ortholog of the well-known chitin receptor LYK5 in *Arabidopsis*, and one a lipoxygenase (LOX) probably involved in biosynthesis of the plant defense-related hormone jasmonic acid, were also up-regulated in the rose response to *B. cinerea* infection (Fig. 2D).

There was a previous report on transcriptome analysis of the rose response to *B. cinerea* infection [28], and thus a cross-comparison of proteomic data in this study and published transcriptomic data was performed. Because only 883 differentially expressed genes (DEGs) encoding putative cell surface receptors, hormone signal related proteins, and transcription factors were publicly available in the transcriptome [28], the cross-comparison was performed based on these available DEGs. After matching with identified rose proteins and DEPs in this study, 26 of these available DEGs were also found in the proteome and there were only two consistent DEGs/DEPs (Supplementary Data Table S3), indicating limited correlation of proteomic and transcriptomic data. The two consistent DEGs/DEPs, including a carboxylesterase that may be involved in gibberellin signaling [37] and an ethylene-responsive transcription factor, were up-regulated at both transcriptional and protein levels (Supplementary Data Table S3).

### PR10 proteins are involved in rose resistance to *B. cinerea*

**Figure 3:**
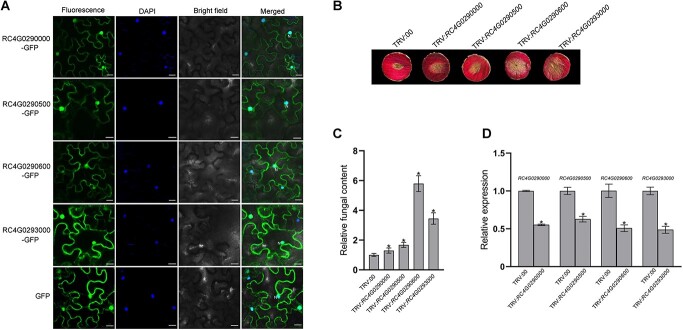
Functional verification of four rose PR10 proteins. **A** Subcellular localization of the four PR10 proteins using *Agrobacterium*-mediated transient expression assay in *N. benthamiana* leaves. N, nucleus. Nuclei were visualized with DAPI. Scale bar = 20 μm. **B** Disease phenotypes of gray mold on rose petal discs treated with TRV:00 (control), TRV:*RC4G0290000*, TRV:*RC4G0290500*, TRV:*RC4G0290600*, and TRV:*RC4G0293000* at 48 hpi. **C** Real-time PCR analysis of relative fungal content in inoculated petal discs at 48 hpi. **D** Relative expression of the four PR10 genes in control (TRV:00) and silenced petal discs at 6 days post-silencing. In **C** and **D**, data are shown as mean ± standard deviation of three biological replicates. ^*^P < 0.05 compared with the control.

Different from most PR proteins, the PR10 protein family, which is a large group, does not contain a signal peptide and is usually intracellular and cytosolic [38]. Four up-regulated rose PR10 proteins—RC4G0290000, RC4G0290500, RC4G0290600, and RC4G0293000—were randomly selected for checking their subcellular localization. Noticeably, they were all localized in the plant cytoplasm as well as the nucleus (Fig. 3A), which is similar to cotton GbPR10.5D1 [39], although they are not orthologs of GbPR10.5D1 (Supplementary Data Fig. S1). To investigate the putative role of PR10 proteins in rose resistance to *B. cinerea*, virus-induced gene silencing (VIGS) in rose petal discs [27] was performed to silence the four PR10 genes. Disease phenotype (Fig. 3B) and fungal content (Fig. 3C) analyses showed that the resistance to *B. cinerea* was attenuated in all silenced petal discs compared with the control petal disc. The silencing efficiency of PR10 genes in the VIGS assay was confirmed by quantitative reverse-transcription PCR (qRT–PCR) analysis (Fig. 3D). These results indicate that PR10 proteins are involved in rose resistance to *B. cinerea*.

**Figure 4:**
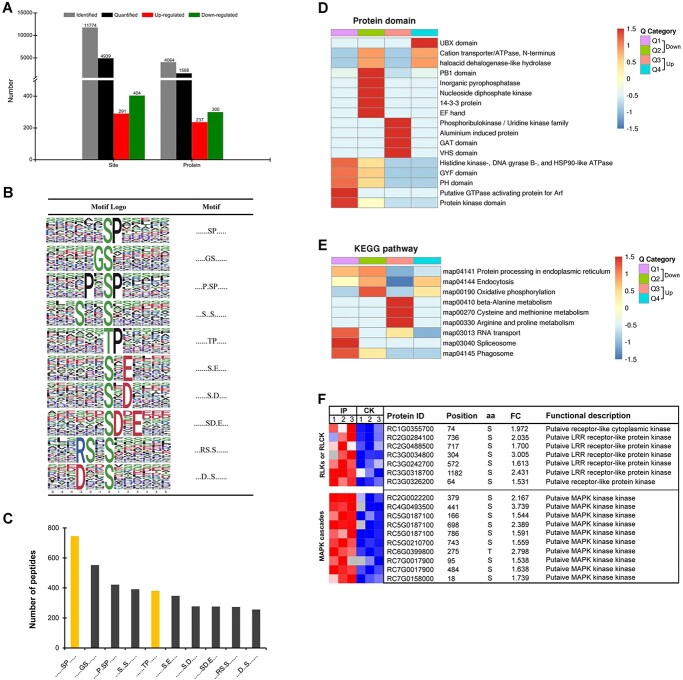
*B. cinerea* infection-regulated rose phosphoproteome. **A** Characterization of phosphorylation sites and phosphoproteins in the phosphoproteome. **B** Graphical representation of top 10 enriched phosphorylation motifs. S or T in the middle indicates the phosphorylated Ser or Thr. **C** Bar chart showing the number of peptides containing each phosphorylation motif shown in **B**. **D** Protein domain enrichment analysis of differentially phosphorylated proteins. **E** KEGG enrichment analysis of differentially phosphorylated proteins. **F** Heat map showing the phosphorylation level of some differentially phosphorylated sites in infected petals (IP) and uninfected petals (CK).

### *Botrytis cinerea* infection-regulated rose phosphoproteome

A total of 11 774 phosphorylation sites in 4064 rose proteins (Supplementary Data Table S4) were identified in our phosphoproteome analysis (Fig. 4A). For these phosphorylation sites in rose proteins, the percentage distributions of Ser phosphorylation, Thr phosphorylation, and Tyr phosphorylation were 88.92, 10.63, and 0.45, respectively (Supplementary Data Fig. S2A), which is comparable to previous phosphoproteome analyses in other plants [40, 41]. Analysis of the numbers of phosphorylation sites in each phosphoprotein showed that most (45.30%, 1841) of 4064 identified rose phosphoproteins have a single phosphorylation site, but there are also up to 511 (12.57%) proteins having six or more phosphorylation sites (Supplementary Data Fig. S2B), of which 25 rose proteins have even 20–39 phosphorylation sites (Supplementary Data Table S5). In addition, a total of 77 phosphorylation motifs were identified and 29 of them were not observed previously in plants (Supplementary Data Table S6). Meanwhile, . . . . . .SP. . . . . and . . . . . .TP. . . . ., which are two well-known motifs phosphorylated by MAPKs [42], also account for a large peptide population (Fig. 4B and C).

**Figure 5:**
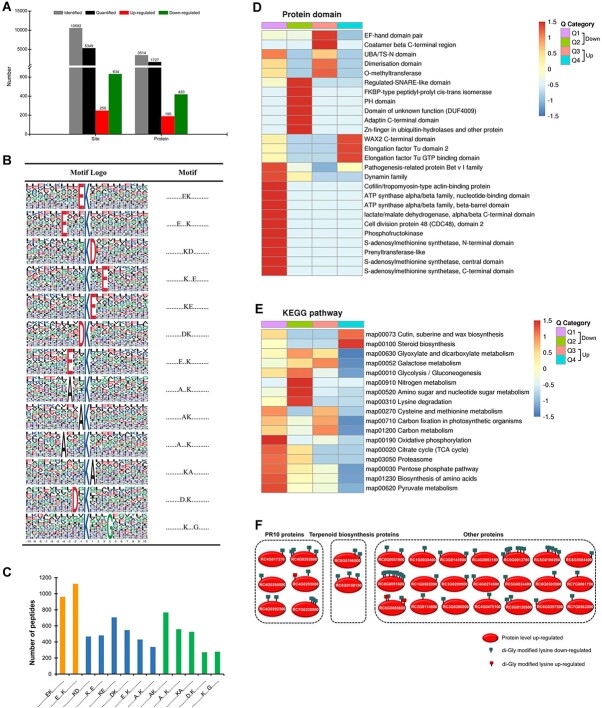
*Botrytis cinerea* infection-regulated rose ubiquitylome. **A** Characterization of ubiquitination sites and ubiquitinated proteins in the rose ubiquitylome. **B** Graphical representation of 13 identified ubiquitination motifs. **C** Bar chart showing the number of peptides containing each ubiquitination motif in **B**. Orange columns and green columns indicate evolutionarily conserved motifs and novel ubiquitination motifs, respectively. **D** Protein domain enrichment analysis of differentially ubiquitinated proteins. **E** KEGG enrichment analysis of differentially ubiquitinated proteins. **F** Schematic representation of 29 rose proteins that showed increased protein abundance along with reduced ubiquitination.

Noticeably, a total of 4939 phosphorylation sites in 1588 rose proteins could be quantified, and 291 phosphorylation sites in 237 rose proteins and 404 phosphorylation sites in 300 rose proteins were up-regulated and down-regulated (FC > 1.5 and *P* value <0.05), respectively, in the rose response to *B. cinerea* infection (Fig. 4A; Supplementary Data Table S7). Protein domain enrichment analysis of differentially phosphorylated proteins showed that UBX domain-containing protein, cation transporter/ATPase, and haloacid dehalogenase-like hydrolase were up-regulated (Fig. 4D). KEGG enrichment analysis indicated that up-regulated phosphorylated proteins were mainly enriched in amino acid metabolic pathways, including ‘beta-alanine metabolism’, ‘cysteine and methionine metabolism’, and ‘arginine and proline metabolism’ (Fig. 4E). Further analysis showed that many rose protein kinases probably involved in plant PTI signaling, including RLKs, RLCK, and MAPK cascades, were up-regulated phosphoproteins (Fig. 4F).

### *Botrytis cinerea* infection-regulated rose ubiquitylome

A total of 10 582 ubiquitination sites in 3514 rose proteins (Supplementary Data Table S8) were identified in our ubiquitylome analysis (Fig. 5A). Although most (43.63%, 1533) of 3514 identified rose proteins have a single ubiquitination site, there are up to 477 (13.57%) proteins having six or more ubiquitination sites (Supplementary Data Fig. S3), of which 18 rose proteins have even 20–34 ubiquitination sites (Supplementary Data Table S9). In addition, a total of 13 ubiquitination motifs (Fig. 5B; Supplementary Data Table S10) for 7450 unique sites which account for 70.40% of all ubiquitination sites were identified. The top two enriched motifs are . . . . . . . . .EK. . . . . . . . . . and . . . . . .E. . .K. . . . . . . . . . (Fig. 5C), which are evolutionarily conserved motifs and have been reported previously in different plant species [43–45]. Meanwhile, five novel ubiquitination motifs, including . . . . . . . . .AK. . . . . . . . . ., . . . . . .A. . .K. . . . . . . . . ., . . . . . . . . . .KA. . . . . . . . ., . . . . . . . .D.K. . . . . . . . . ., and . . . . . . . . . .K. . .G. . . . . ., were also identified in the ubiquitylome (Fig. 5B and C).

Among 5349 quantified ubiquitination sites, 250 ubiquitination sites in 190 rose proteins and up to 634 ubiquitination sites in 420 rose proteins were up-regulated and down-regulated (FC > 1.5 and *P* value <0.05), respectively (Fig. 5A; Supplementary Data Table S11). A volcanic map of these differentially ubiquitinated sites showed that the fold change of down-regulated ubiquitinated sites was overall higher than that of up-regulated ubiquitinated sites (Supplementary Data Fig. S4), indicating that global ubiquitination levels decreased in the rose response to *B. cinerea* infection. Protein domain enrichment analysis showed that PR10 protein, ATP synthase, cell division protein 48 (CDC48), and *S*-adenosylmethionine synthetase were down-regulated ubiquitinated proteins (Fig. 5D). KEGG enrichment analysis indicated that down-regulated ubiquitinated proteins were mainly enriched in some energy-producing primary metabolic pathways, including ‘biosynthesis of amino acids’, ‘citrate cycle (TCA cycle)’, ‘glycolysis/gluconeogenesis’, and ‘amino sugar and nucleotide sugar metabolism’ (Fig. 5E).

**Figure 6:**
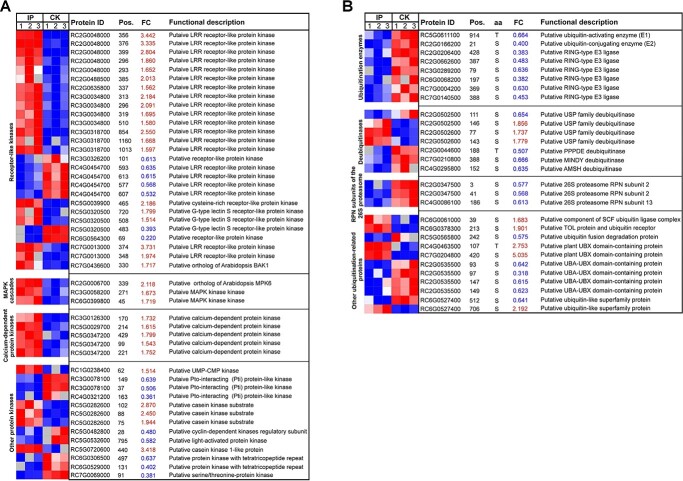
Display of differentially ubiquitinated kinases and differentially phosphorylated ubiquitination-related proteins. **A** Heat map showing the ubiquitination level of ubiquitination sites in protein kinases in infected petals (IP) and uninfected petals (CK). **B** Heat map showing the phosphorylation level of phosphorylation sites in ubiquitination-related proteins in IP and CK. Shades of red and blue in the fold change (FC) column indicate up- and down-regulation, respectively. Noticeably, many protein kinases have multiple ubiquitination sites and ubiquitination-related proteins have multiple phosphorylation sites.

**Figure 7:**
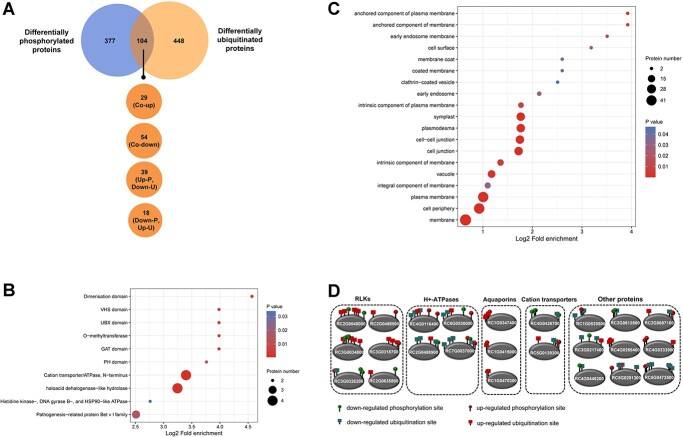
Characterization of co-regulated rose proteins by both phosphorylation and ubiquitination. **A** Venn diagram of differentially phosphorylated proteins and differentially ubiquitinated proteins. Co-up, common up-regulation; Co-down, common down-regulation; Up-P and Down-U, up-regulation in phosphorylation and down-regulation in ubiquitination; Down-P and Up-U, down-regulation in phosphorylation and up-regulation in ubiquitination. **B** Protein domain enrichment analysis of co-regulated rose proteins. **C** GO enrichment analysis of co-regulated rose proteins and the enriched ‘cellular component’ category. **D** Schematic representation of 25 co-regulated plasma membrane proteins.

A cross-comparison of the ubiquitylome and proteome indicated that a total of 29 rose proteins showed increased protein abundance along with reduced ubiquitination, and 15 rose proteins showed decreased protein abundance along with increased ubiquitination (Supplementary Data Fig. S5; Supplementary Data Table S12). Further analysis showed that multiple ubiquitination sites of some rose proteins were uniformly down-regulated (Fig. 5F). Among 12 up-regulated rose PR10 proteins (Fig. 2D), half of them also showed reduced ubiquitination (Fig. 5F), indicating that *B. cinerea* infection resulted in decreased ubiquitination and enhanced protein abundance in these PR10 proteins. In addition, two rose proteins probably involved in the biosynthesis of terpenoids, which were shown to be involved in rose petal resistance against *B. cinerea* infection [28], also showed increased protein abundance along with reduced ubiquitination (Fig. 5F).

### Identification of differentially ubiquitinated kinases and differentially phosphorylated ubiquitination-related proteins

The ubiquitination of kinases and the phosphorylation of ubiquitination-related proteins, especially E3 ligases, are an important embodiment of phosphorylation and ubiquitination crosstalk in plant immunity [1]. Thus, we tried to identify differentially ubiquitinated kinases and differentially phosphorylated ubiquitination-related proteins in the rose response to infection with *B. cinerea*. Noticeably, up to 28 rose protein kinases, many of which have multiple ubiquitination sites, were differentially ubiquitinated in infected rose petals (Fig. 6A). Most (42.86%, 12) of these differentially ubiquitinated kinases belong to RLKs that are probably involved in plant PTI signaling (Fig. 6A). Among them, one rose RLK (RC7G0436600) is the ortholog of *Arabidopsis* BAK1, which is a key co-receptor of plant PTI [46]. In addition, some members of MAPK cascades, of which one is the ortholog of *Arabidopsis* plant immunity regulator MPK6 [47], calcium-dependent protein kinases, which regulate plant immunity by activating, calcium signaling [48], and other protein kinases were also differentially ubiquitinated (Fig. 6A). On the other hand, a lot of ubiquitination enzymes, deubiquitinases, RPN subunits of the 26S proteasome, and other ubiquitination-related proteins were also differentially phosphorylated in the rose response to *B. cinerea* infection (Fig. 6B). Among these differentially (uniformly down-regulated) phosphorylated ubiquitination enzymes, E3 ubiquitin ligases were dominant (Fig. 6B), indicating possible crosstalk between kinases and E3 ubiquitin ligases in the rose response to *B. cinerea* infection.

### Identification of co-regulated proteins by both phosphorylation and ubiquitination

A cross-comparison of the phosphoproteome and ubiquitylome showed that there were a total of 1640 rose proteins that have both phosphorylation and ubiquitination sites (Supplementary Data Fig. S6). Further comparative analysis of differentially phosphorylated proteins and differentially ubiquitinated proteins showed that both of two PTMs of 104 proteins were dynamically regulated in rose upon *B. cinerea* infection (Fig. 7A; Supplementary Data Table S13). Among these proteins co-regulated by both phosphorylation and ubiquitination, common up-regulation and down-regulation could be observed in 29 and 54 rose proteins, respectively (Fig. 7A). Protein domain enrichment analysis showed that cation transporter/ATPase, haloacid dehalogenase-like hydrolase, and PR10 protein were dynamically regulated by both phosphorylation and ubiquitination (Fig. 7B). Further analysis showed that three PR10 proteins were co-regulated by both phosphorylation and ubiquitination (Supplementary Data Table S13). Gene Ontology (GO) enrichment analysis of these co-regulated proteins showed that membrane-associated subcategories were dominant in the ‘cellular component’ category (Fig. 7C). The plant plasma membrane acts as the primary interface of plant–microbe interaction and plasma membrane proteins play a key role in triggering plant immunity, especially for plant PTI [49, 50]. Noticeably, up to 24 rose plasma membrane proteins, including six RLKs, four H^+^-ATPases, three aquaporins, two cation transporters, and nine other plasma membrane proteins, were co-regulated, and many of them had multiple regulated sites (Fig. 7D; Supplementary Data Table S14). These results indicate that phosphorylation and ubiquitination mainly converge on the plant plasma membrane in the rose response to infection with *B. cinerea*.

### Functional verification of a plasma membrane aquaporin in rose resistance to *B. cinerea*

**Figure 8:**
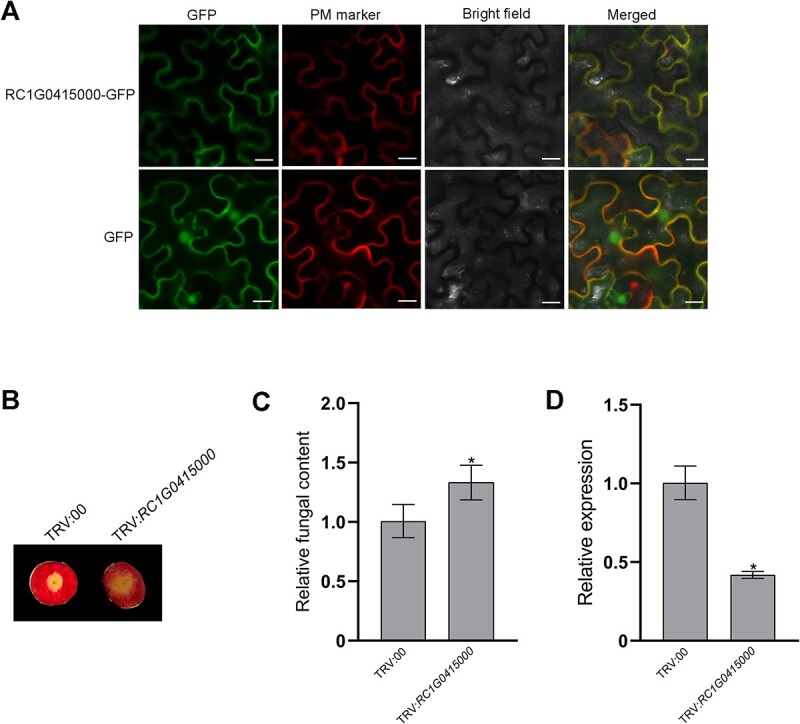
Functional verification of a rose aquaporin RC1G0415000. **A** GFP fluorescence in *N. benthamiana* leaves expressing the RC1G0415000-GFP fusion protein or GFP (control). Plasma membrane (PM) marker AtPIP2A-mCherry was also expressed simultaneously in *N. benthamiana* leaves. Scale bar = 20 μm. **B** Disease phenotypes of gray mold on petal discs treated with TRV:00 (control) and TRV:*RC1G0415000* at 48 hpi. **C** Real-time PCR analysis of relative fungal content in inoculated petal discs at 48 hpi. **D** Relative expression of *RC1G0415000* in control (TRV:00) and silenced (TRV:*RC1G0415000*) petal discs at 6 days post-silencing. In **C** and **D** data are shown as mean ± standard deviation of three biological replicates. *P < 0.05 compared with the control.

Aquaporins are important membrane channel proteins and recent reports highlight the involvement of plasma membrane aquaporins in plant disease resistance [51, 52]. Thus, one (RC1G0415000) of three co-regulated rose aquaporins (Fig. 7D) was selected for functional verification. As expected, a subcellular localization assay showed that RC1G0415000 was localized in the plant plasma membrane (Fig. 8A). Moreover, a VIGS assay was also performed to investigate the putative role of RC1G0415000 in rose disease resistance. Disease phenotype (Fig. 8B) and fungal content (Fig. 8C) analyses showed that the resistance to *B. cinerea* was attenuated in *RC1G0415000*-silenced petal discs compared with the control petal disc. The silencing efficiency of *RC1G0415000* in the VIGS assay was confirmed by qRT–PCR analysis (Fig. 8D). Collectively, the results show that plasma membrane aquaporin RC1G0415000 is involved in rose resistance to *B. cinerea*.

## Discussion

### Our proteomic analysis increases the database of phosphorylation and ubiquitination sites in plants

Proteomics enables a systematic investigation of the abundance and PTMs of proteins, which are the ultimate executors of biological functions, and greatly accelerates plant research [53]. Ornamental plants are an important component of agriculture and horticulture and great progress has occurred in ornamental plant genomics [54]. However, proteomics analysis in ornamental plants remains relatively unexplored. An integrated analysis of the proteome, phosphoproteome, and ubiquitylome in rose petals upon *B. cinerea* infection was performed in this study. There are challenges in plant proteomics, such as an inability to measure the entire proteome and technical limitations in quantitation procedures [55]. To acquire more accurate results in our proteomic analysis, proteome, phosphoproteome, and ubiquitylome analyses were performed with the same samples, and phosphorylation and ubiquitination quantifications in phosphoproteome and ubiquitylome were normalized by protein quantifications to eliminate the interference caused by protein abundance differences in the proteome, as previously reported [56]. Noticeably, our rose proteome identified a total of 6165 proteins, which is more than that in the proteomes of other ornamental plants, including petunias [43], daylily [57], and chrysanthemum [58]. In addition, >10 000 phosphorylation or ubiquitination sites were identified in both the phosphoproteome and ubiquitylome of rose, and the number of phosphorylation or ubiquitination sites was significantly more than in other published PTM studies in plants [43, 59–62]. Meanwhile, analysis of sequence properties of phosphorylated and ubiquitinated proteins showed that up to 77 phosphorylation motifs and 13 ubiquitination motifs were identified in our phosphoproteome and ubiquitylome, respectively. Many of them were novel phosphorylation or ubiquitination motifs, which will contribute to the exploration of novel kinase–substrate interactions and ubiquitin ligase–substrate interactions. Our proteomic analysis increases the database of phosphorylation and ubiquitination sites in plants, and also provides a valuable resource for future studies on rose, which has high-quality reference sequences and represents an ideal model species for ornamental plants [24].

### Important contribution of PR10 proteins to the rose response to *B. cinerea* infection

Among 17 known PR protein families, PR10 proteins belong to a multigene family and usually have antimicrobial and ribonuclease activities [38, 63]. A lot of PR10 genes were significantly up-regulated in the plant response to pathogen infection at the transcriptional or protein level and the function of PR10 proteins in plant resistance to pathogen infection has been verified in different plant species [39, 63–66]. Function annotation of rose DEPs showed that up to 12 PR10 proteins were uniformly up-regulated upon *B. cinerea* infection and four of them were selected for functional verification. Noticeably, VIGS assay showed that all of them were involved in the resistance of rose petals to *B. cinerea* (Fig. 3). These results highlight the important contribution of PR10 proteins to the rose response to infection with *B. cinerea.*

It has been reported that PR10 proteins are phosphoproteins and phosphorylation of PR10 proteins increases their ribonuclease activity, which is related to their antimicrobial activity, thus contributing to plant disease resistance [38]. However, nothing is known about other PTMs and PTM crosstalk in PR10 proteins. Here, phosphoproteome and ubiquitylome analyses indicated that six PR10 proteins showed increased protein abundance along with reduced ubiquitination and three PR10 proteins were co-regulated by both phosphorylation and ubiquitination, indicating the presence of ubiquitination and PTM crosstalk in PR10 proteins. Co-regulation of a ribonuclease by phosphorylation and ubiquitination was reported [67], and thus we infer that co-regulation of PR10 proteins by phosphorylation and ubiquitination may fine-tune their ribonuclease activity in the rose response to infection with *B. cinerea*.

### Important contribution of plant pattern-triggered immunity signaling in the rose response to *B. cinerea* infection

Upon the recognition between plant PRRs and pathogen-associated molecular patterns (PAMPs), plant PTI immune signaling events, such as ROS burst, altered ion fluxes, MAPK cascades, and transcriptional programming, will be initiated and effectively control the colonization of pathogenic fungi [6, 68]. RLKs are an essential component of the plasma membrane-associated receptor complexes during PTI [69], and *B. cinerea* infection significantly induced the expression or phosphorylation of some rose RLKs. RLCKs, which are phosphorylated by PRRs and the downstream MAPK cascades, are also important components of plant PTI signaling [6], and *B. cinerea* infection significantly induced the phosphorylation of one RLCK and seven members of MAPK cascades (Fig. 4F). These immunity-related protein kinases, including RLKs, RLCKs, and MAPK cascades, were proven to regulate plant resistance to *B. cinerea* [20, 21]. Thus, our results highlight the important contribution of plant PTI signaling in the rose response to infection with *B. cinerea*. Among the two up-regulated rose RLKs (Fig. 2D), one is the ortholog of *Arabidopsis* LYK5, which is a major receptor for recognizing chitin, a well-known fungus PAMP [70], indicating conserved components of plant PTI signaling in different plant species.

*Arabidopsis* BIK1 (*Botrytis*-induced kinase1) is a well-known RLCK regulating plant resistance to *B. cinerea* [71] and was proven to show phosphorylation and ubiquitination modification [72, 73]. RC1G0449700, the ortholog of *Arabidopsis* BIK1, was also identified in our rose phosphoproteome and ubiquitylome (Supplementary Data Tables S4 and S8), which proves its phosphorylation and ubiquitination modification. However, most phosphorylation and ubiquitination sites of RC1G0449700 were only identified in infected petals but not uninfected petals (control). These phosphorylation and ubiquitination sites, which were identified in at least two of three replicates in infected and uninfected petals, were taken into account in fold change analysis in this study, and thus RC1G0449700, the ortholog of *Arabidopsis* BIK1, was not classified as a differentially phosphorylated or ubiquitinated protein. Meanwhile, we find that some other RLCKs and RLKs show similar phenomena (Supplementary Data Tables S4 and S8). Thus, these RLCKs and RLKs, which were identified in our rose phosphoproteome and ubiquitylome but were not classified as differentially phosphorylated or ubiquitinated proteins, are also worthy of further investigation.

### Crosstalk between RLKs and E3 ubiquitin ligases in the rose response to *B. cinerea* infection

A lot of differentially ubiquitinated kinases and differentially phosphorylated ubiquitination-related proteins were identified in rose upon *B. cinerea* infection. Among these differentially ubiquitinated kinases and differentially phosphorylated ubiquitination enzymes, RLKs, including the ortholog of *Arabidopsis* BAK1, and E3 ubiquitin ligases were dominant (Fig. 6). Crosstalk between RLKs and E3 ubiquitin ligases in plant PTI has been identified in several different plant species, and E3 ubiquitin ligase-mediated ubiquitination of RLKs usually causes their degradation or changes their subcellular localization, and RLK-mediated phosphorylation of E3 ubiquitin ligases usually affects their patterns of interaction with substrates or enzymatic activity [1]. Thus, the conserved crosstalk between RLKs and E3 ubiquitin ligases may be an important embodiment of phosphorylation and ubiquitination crosstalk in the rose response to infection with *B. cinerea*. Further analysis showed that most of these rose RLKs were up-regulated by ubiquitination and all rose E3 ubiquitin ligases were down-regulated by phosphorylation (Fig. 6). Uncontrolled immune responses impair plant growth and development and there are rate-limiting components, including BAK1, in plant PTI [74, 75]. Phosphorylation and ubiquitination were proven to regulate rate-limiting component-mediated immune homeostasis [74]. Thus, increased ubiquitination of rose RLKs and decreased phosphorylation of rose E3 ubiquitin ligases may contribute to immune homeostasis rate-limiting components in the rose response to *B. cinerea* infection.

### Phosphorylation and ubiquitination converge on PTI signaling components in the plant plasma membrane

In addition, many other different classes of kinases and ubiquitination-related proteins were also differentially ubiquitinated and phosphorylated, respectively, and a total of 104 proteins, including the previously mentioned PR10 proteins, were co-regulated by both phosphorylation and ubiquitination. These results provide valuable information for revealing new aspects of phosphorylation and ubiquitination crosstalk in plant disease resistance. Among these co-regulated proteins, plasma membrane proteins were dominant and six RLKs, four H^+^-ATPases, three aquaporins, two cation transporters, and nine other plasma membrane proteins, were co-regulated in rose upon *B. cinerea* infection (Fig. 7D). Co-regulation of RLKs can be interpreted as the previously mentioned crosstalk between RLKs and E3 ubiquitin ligases. In addition to RLKs, H^+^-ATPases and aquaporins have proven to be important regulators of plant PTI [52, 76, 77]. Plasma membrane H^+^-ATPases (AHAs), which are an important ion pump and pump out protons from the cytosol into the extracellular space, negatively regulate membrane depolarization and apoplastic alkalization, which are important cellular responses during PTI [76]. PAMP treatment results in dramatic changes (down-regulation) in the phosphorylation status of H^+^-ATPases and phosphorylation of H^+^-ATPases negatively impacts their AHA activity [76]. Because the four H^+^-ATPases were not differentially expressed in the proteome (Supplementary Data Table S1), ubiquitination of H^+^-ATPases may not directly affect their degradation in the rose response to infection with *B. cinerea*. We infer that co-regulation of H^+^-ATPases by phosphorylation and ubiquitination may fine-tune their AHA activity, thus regulating membrane depolarization and apoplastic alkalization during rose PTI signaling. Aquaporins are membrane channel proteins and can transport apoplastic hydrogen peroxide (H_2_O_2_) into the cytoplasm during plant PTI signaling [52, 77]. Phosphorylation was shown to regulate gating and trafficking of aquaporins and promotes H_2_O_2_ transport in plant immunity [52, 77]. Therefore, co-regulation of aquaporins by phosphorylation and ubiquitination may fine-tune H_2_O_2_ transport during rose PTI signaling. In addition, RC1G0539500 in other plasma membrane proteins (Supplementary Data Table S14) is a putative remorin protein that is also involved in PTI [78]. Remorin is an important component of membrane nanodomains and was proven to regulate actin remodeling during plant PTI signaling by modulating formin condensation [78]. Remorins usually undergo phosphorylation, and phosphorylation is required for their function in plant immunity [79]. Thus, co-regulation of remorin by phosphorylation and ubiquitination may fine-tune actin remodeling during rose PTI signaling. Because H^+^-ATPase, aquaporin, and remorin can be the substrates of plant RLKs [80, 81], all of them are also PTI signaling components in the plant plasma membrane and may be directly regulated by plasma membrane-associated receptor complexes (Supplementary Data Fig. S7). Cation transporters mediate cation transport across the plant plasma membrane, but little is known about their role in plant immunity [82]. Co-regulation of cation transporters by phosphorylation and ubiquitination may fine-tune cation transport, especially calcium (Ca^2+^) transport, because Ca^2+^ elevation in the cytosol also represents a hallmark of early PTI immune responses [83].

Overall, our study highlights the important involvement of PR10 proteins and PTI signaling in the rose response to infection with *B. cinerea*. Moreover, close interactions between phosphorylation and ubiquitination were observed and possible crosstalk between RLKs and E3 ubiquitin ligases and co-regulation of PTI signaling components in the plant plasma membrane are important embodiments of interactions between phosphorylation and ubiquitination in the rose response to *B. cinerea* infection (Supplementary Data Fig. S7). Our results noticeably advance our understanding of the mechanisms underlying rose resistance to *B. cinerea* and also PTM crosstalk in plant immunity. The specific mechanisms by which phosphorylation and ubiquitination crosstalk regulate rose resistance to *B. cinerea* remain to be investigated in the future.

## Materials and methods

### Sample preparation for proteomics

Petals of healthy rose (*Rosa hybrida*) cv. ‘Carola’, which were purchased from a commercial grower, were inoculated with a *B. cinerea* strain as described previously [28]. For inoculation, rose petals were wounded by making punctures and then were inoculated with 1-μl drops of *B. cinerea* conidia *(*10^5^ spores ml^−1^) on each wound. At 24 hpi, inoculated petal regions were sampled. To obtain uninfected petals (control), rose petals were inoculated with buffer and were also sampled at 24 hpi with buffer. These infected and uninfected rose samples were used for subsequent proteomics analysis in this study.

### Cytological observation of *B. cinerea* growth in infected rose petals

To check whether *B. cinerea* had infected rose petals at 24 hpi, cytological analysis of *B. cinerea* growth were performed using wheat germ agglutinin (WGA) conjugated to Alexa 488 (Invitrogen, Carlsbad, CA, USA) as described previously [84]. Infected rose petals at 24 hpi were cut into small pieces of 2–3 cm^2^, which were then fixed and decolorized in ethanol/acetic acid (1:1, v/v) for several days. Finally, these tissues were stained with 0.02 g l^*−*1^ WGA-Alexa 488 and were observed under a confocal fluorescence microscope (TCS SP8, Leica, Germany).

### Workflow of proteome, phosphoproteome, and ubiquitylome analyses

To investigate proteome, phosphorylation, and ubiquitination changes in rose petals upon *B. cinerea* infection, proteome, phosphoproteome, and ubiquitylome analyses of infected petals and uninfected petals were performed with three biological replications and carried out by PTM Biolabs (Hangzhou, China). For proteomic analysis, protein extraction, tryptic digestion, 4D label-free, liquid chromatography–tandem mass spectrometry (LC–MS/MS) analysis, database search, and bioinformatics analysis were performed sequentially. IMAC and anti-K-ε-GG antibody were used for enrichment of phosphorylated and ubiquitinated peptides in phosphoproteome and ubiquitylome, respectively.

### Protein extraction

Protein extraction for proteome, phosphoproteome, and ubiquitylome analyses was performed as described previously [45]. After grinding, samples of infected and uninfected petals were placed in lysis buffer containing 10 mM dithiothreitol, 1% protease inhibitor, 1% phosphatase inhibitor, and 50 μM PR-619 (a general inhibitor of deubiquitylating enzymes) for sonication. The lysed samples were centrifuged to obtain the supernatant, to which was added 0.1 M ammonium acetate/methanol for overnight precipitation. Finally, precipitated proteins were dissolved with 8 M urea, and the BCA Protein Assay Kit (Beyotime Biotechnology, Shanghai, China) was used to determine the protein concentration.

### Tryptic digestion

The same amounts of protein solutions from infected and uninfected petals were used for tryptic digestion. Twenty percent trichloroacetic acid was slowly added to the protein solutions, which were then precipitated and centrifuged. Precipitate was washed with pre-cooled acetone and diluted by adding 200 mM tetraethylammonium bromide. Trypsin (1:50) was added to the protein solutions for overnight digestion. Finally, the tryptic peptides were reduced with 5 mM dithiothreitol and then alkylated by adding 11 mM iodoacetamide.

### Affinity enrichment of phosphorylated peptides

To enrich phosphorylated peptides, tryptic peptides were dissolved in buffer containing 50% acetonitrile/6% trifluoroacetic acid, and the supernatant was added to pre-washed resin containing IMAC for incubation. The resin containing IMAC was then washed with buffer containing 50% acetonitrile/6% trifluoroacetic acid/0.1% trifluoroacetic acid, and bound peptides were eluted by adding 10% ammonia. Finally, bound peptides were cleaned by using C18 ZipTips (Millipore, Burlington, MA, USA), and cleaned peptides were vacuum-dried.

### Affinity enrichment of ubiquitinated peptides

To enrich Lys ubiquitination (Kub) peptides, tryptic peptides were dissolved in NETN buffer containing 100 mM NaCl/1 mM EDTA/50 mM Tris–HCl/0.5% NP-40, and pre-washed ubiquitination resin PTM-1104 (PTM Biolabs, Hangzhou, China), which contains anti-K-ε-GG antibody, was added to the supernatant for overnight incubation. Peptides were washed with NETN buffer and dH_2_O_2_ and then eluted by using 0.1% trifluoroacetic acid. Finally, bound peptides were also cleaned by using C18 ZipTips, and cleaned peptides were vacuum-dried.

### LC–MS/MS analysis

Peptides of rose samples in this study were dissolved in solvent A containing 0.1% formic acid/2% acetonitrile and then separated using a reverse-phase analytical column (75 μm inner diameter and 15 cm length) coupled to the NanoElute ultra-high performance liquid phase system. The gradient of solvent B, containing 0.1% formic acid/100% acetonitrile, consisted of an increase from 6 to 24% over 70 min, 24 to 35% in 14 min, climbing to 80% in 3 min, and holding at 80% for the last 3 min. The peptides were analyzed with a timsTOF Pro mass spectrometer. The electrospray voltage was set at 1.7 kV and the scan range of secondary mass spectrometry was set as *m*/*z* 400–1500. Data acquisition of secondary mass spectrometry was performed using the parallel accumulation–serial fragmentation (PASEF) [85] method.

### Database search and bioinformatic analysis

The database search was performed as previously described [57]. The MS/MS data from proteome, phosphoproteome, and ubiquitylome analyses were processed using the Andromeda search engine in Max Quant (v1.6.15.0) and were searched against the *Rosa chinensis* UniProt database (39 669 sequences). To eliminate the effect of contaminated proteins, trypsin/P was specified as the cleavage enzyme, which allows up to two missing cleavages. The maximum number of peptide modifications was 5 and the minimum length of peptide segment was 7. Carbamidomethylation of cysteine was specified as the fixed modification. In the phosphoproteome analysis, phosphorylation of serine, threonine, and tyrosine, protein N-acetylation, oxidation of methionine, and deamidation of asparagine and glutamine were specified as variable modifications. In the ubiquitylome analysis, lysine ubiquitination, protein N-acetylation, and oxidation of methionine were specified as variable modifications.

The criterion for identifying DEPs and differentially phosphorylated or ubiquitinated sites was FC > 1.5 and *P* value < 0.05. Proteins and phosphorylation or ubiquitination sites that were identified in at least two of three replicates were taken into account in fold change analysis. DEPs and differentially phosphorylated or ubiquitinated sites were divided into four clusters (Q1–Q4) based on fold change, and Q1, Q2, Q3, and Q4 indicate FC < 0.5, 0.5 ≤ FC < 0.667, 2 ≥ FC > 1.5, and FC > 2, respectively. Motif-X software (http://meme-suite.org/tools/momo) was used for identifying phosphorylation and ubiquitination motifs. InterPro (http://www.ebi.ac.uk/interpro/) was used for protein domain enrichment analysis. The KEGG database was used to annotate protein pathways, and KEGG and GO enrichment analyses were performed with the Perl module. Phylogenetic analysis was performed using MEGA5 software. Heat maps in this study were generated with Morpheus (https://software.broadinstitute.org/morpheus/).

### Subcellular localization in *Nicotiana benthamiana*

To investigate the subcellular localization of selected rose proteins, *Agrobacterium tumefaciens*-mediated transient expression was performed in the model plant *N. benthamiana* as described previously [84]. PR10 genes and the aquaporin gene *RC1G0415000* with their natural promoters were cloned into the pGreen (with a C-terminal GFP) vector and then these recombinant plasmids were introduced into *A. tumefaciens* strain GV3101. *Agrobacterium tumefaciens* suspensions containing recombinant plasmids at an OD600 of 0.8–1.0 were infiltrated into *N. benthamiana* leaves. Finally, fluorescence in the infiltrated *N. benthamiana* leaves was examined with a confocal fluorescence microscope (TCS SP8, Leica, Wetzlar, Germany)*.* DAPI (4′,6-diamidino-2-phenylindole dihydrochloride; Solarbio, Beijing, China) was used for staining nuclei and the plasma membrane marker AtPIP2A-mCherry [86] was used as a reference for plasma membrane localization.

### VIGS in rose petals

To silence genes in rose petals, a tobacco rattle virus (TRV)-mediated gene silencing assay was performed as described previously [27]. Specific primers (Supplementary Data Table S15) were designed and fragments were cloned into the TRV vector pTRV2, and introduced into *A. tumefaciens.* Petal discs which were 15-mm in diameter were infiltrated with *A. tumefaciens* cells by vacuum infiltration as described previously [27]*.* At 6 days after infiltration with *Agrobacterium* cells carrying TRV constructs, these petal discs were then inoculated with drops of *B. cinerea* conidia (10^5^ spores ml^*−*1^) as described above.

### Fungal biomass by real-time PCR

For measuring fungal biomass in rose petals inoculated with *B. cinerea*, real-time PCR analysis was performed based on their genomic DNA. Specifically, relative quantification of a cutinase gene (accession number Z69264) from *B. cinerea* and a glyceraldehyde-3-phosphate dehydrogenase (GAPDH) gene (accession number XM_024318078) from rose was performed as previously described [87].

### qRT–PCR analysis

To check the silencing efficiency of rose genes in the VIGS assay, qRT–PCR analysis was performed. Specific primers (Supplementary Data Table S15) were designed and the CFX Connect Real-Time System (Bio-Rad, Richmond, California, USA) was used in this study. The rose housekeeping gene *RhUBI2* [26] was used as an endogenous control, and the 2^−△△CT^ method was used for calculating the expression levels of genes.

## Supplementary Material

Web_Material_uhad238
