# Supplementary material for: Integrated proteomic analysis reveals interactions between phosphorylation and ubiquitination in rose response to Botrytis infection
Source: Hortic Res. 2023 Nov 14;11(1):uhad238. doi: 10.1093/hr/uhad238 (PMC10782497; doi:10.1093/hr/uhad238)
Supplement: Web_Material_uhad238 [file web_material_uhad238.zip › Supplemental Figure S1.docx]

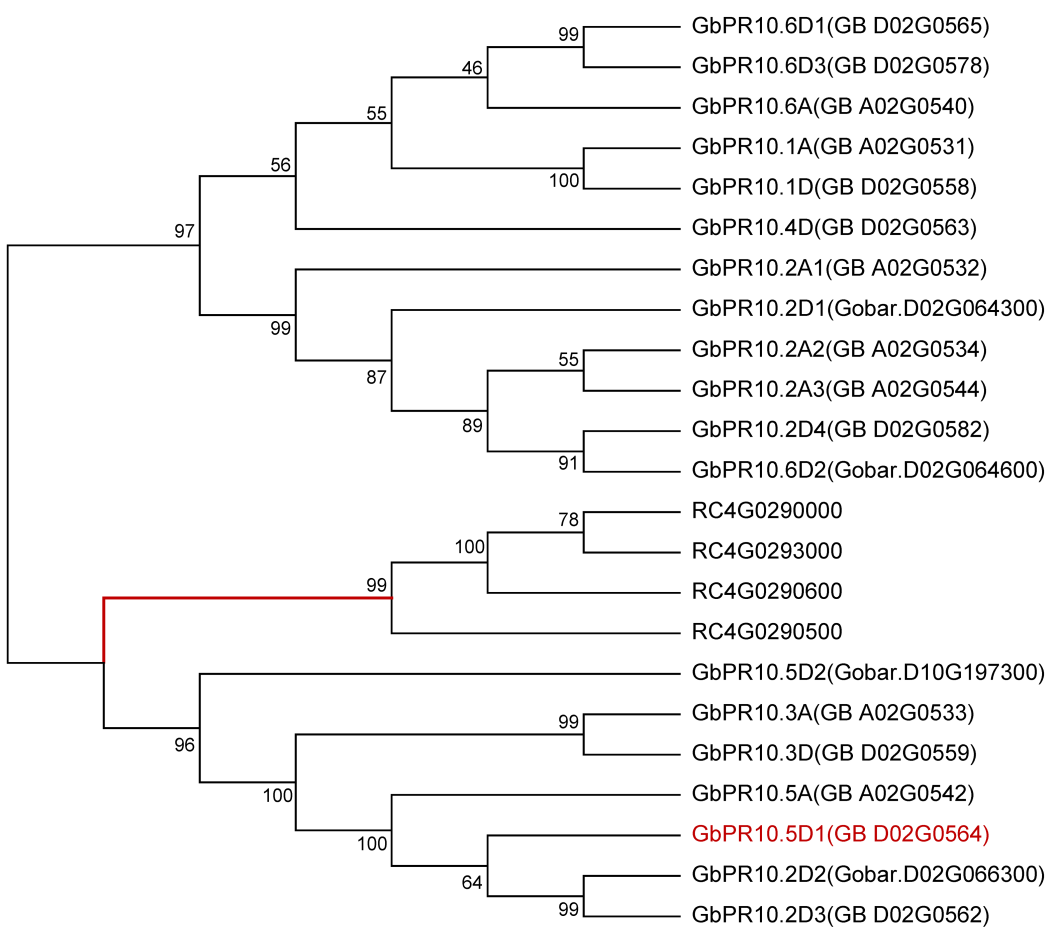


**Supplemental Figure S1** Phylogenetic analysis of four selected rose PR10 proteins (RC4G0290000, RC4G0290500, RC4G0290600, and RC4G0293000) and 19 PR10 proteins from cotton (Gossypium barbadense L.) including GbPR10.5D1. The phylogenetic tree was generated by the MEGA5 software. The neighbor-joining method was used and bootstrap values from 1000 replications were shown.
