# Supplementary material for: Integrated proteomic analysis reveals interactions between phosphorylation and ubiquitination in rose response to Botrytis infection
Source: Hortic Res. 2023 Nov 14;11(1):uhad238. doi: 10.1093/hr/uhad238 (PMC10782497; doi:10.1093/hr/uhad238)
Supplement: Web_Material_uhad238 [file web_material_uhad238.zip › Supplemental Figure S5.docx]

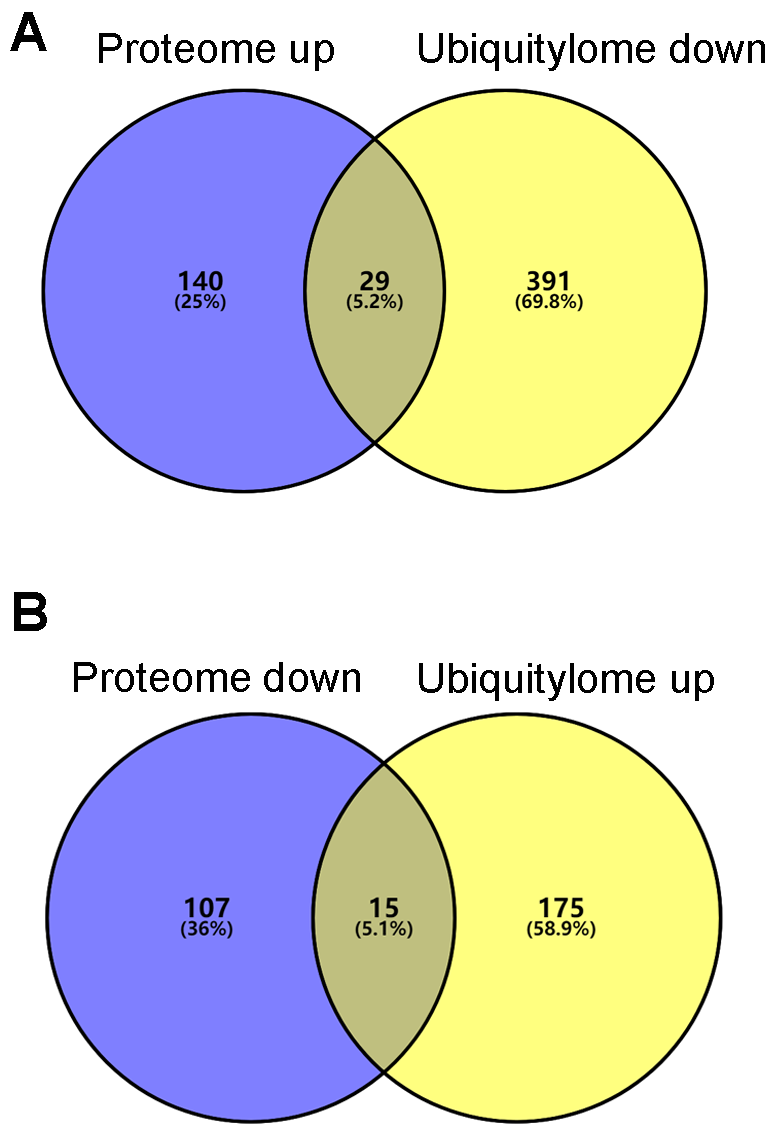


**Supplemental Figure S5** Venn diagram of DEPs and differentially ubiquitinated proteins. (**A**) Venn diagram of up-regulated proteins and down-regulated ubiquitinated proteins. (**B**) Venn diagram of down-regulated proteins and up-regulated ubiquitinated proteins.
