# Supplementary material for: Integrated proteomic analysis reveals interactions between phosphorylation and ubiquitination in rose response to Botrytis infection
Source: Hortic Res. 2023 Nov 14;11(1):uhad238. doi: 10.1093/hr/uhad238 (PMC10782497; doi:10.1093/hr/uhad238)
Supplement: Web_Material_uhad238 [file web_material_uhad238.zip › Supplemental Figure S7.docx]

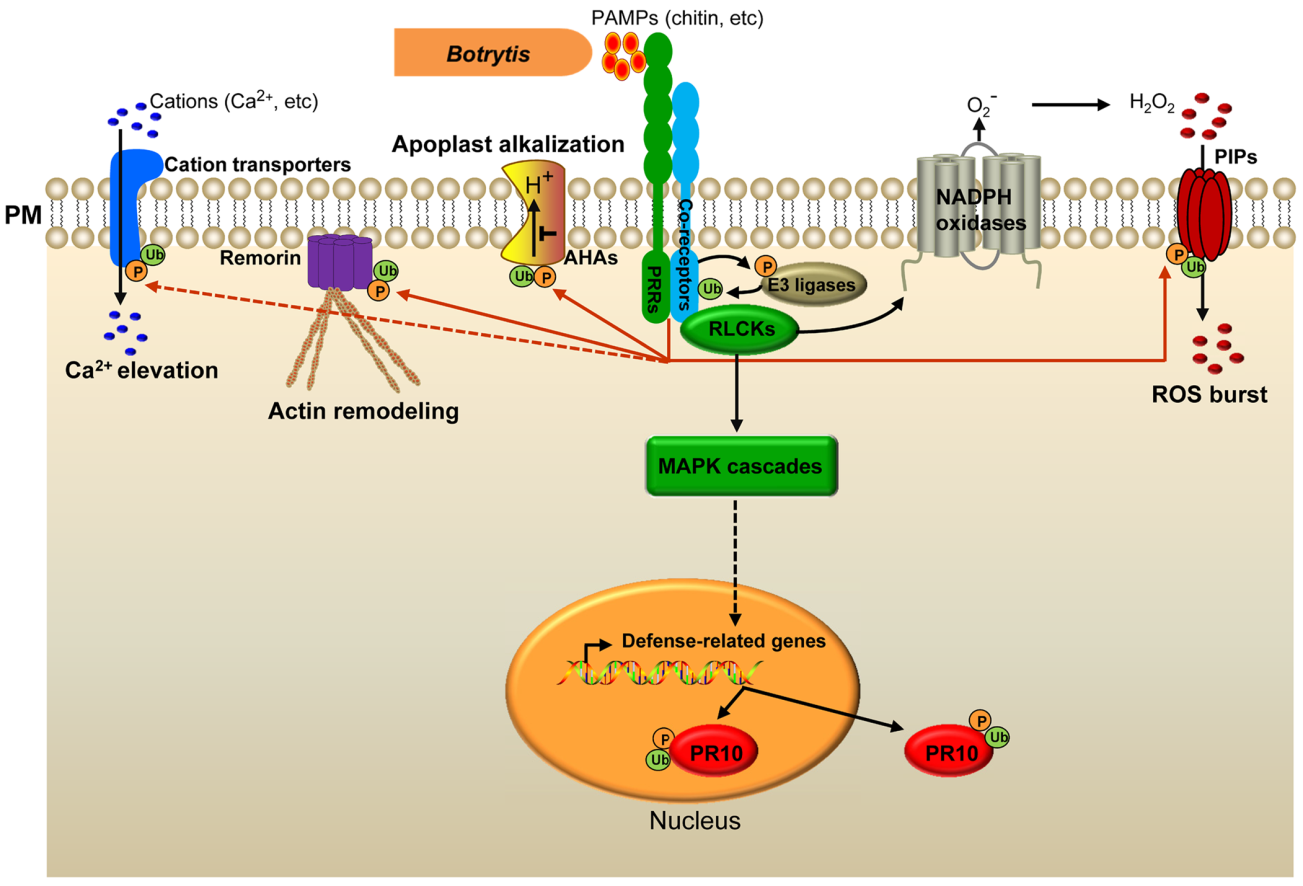


**Supplemental Figure S7** Schematic overview of possible interactions between phosphorylation and ubiquitination in rose response to *B. cinerea* infection. Co-regulation of putative PTI signaling components in plant plasma membrane (PM) including H+-ATPases (AHAs), plasma membrane intrinsic protein (PIPs) belonging to a subfamily of aquaporins, remorin, and cation transporters, may fine-tune corresponding immune signaling events, including apoplastic alkalization, ROS burst, actin remodeling, and Ca^2+^ elevation. In addition, possible crosstalk between RLKs and E3 ligases may contribute to immune homeostasis, and co-regulation of PR10 proteins by phosphorylation and ubiquitination may fine-tune their ribonuclease activity.
