# Supplementary figures and images for: Integrated proteomic analysis reveals interactions between phosphorylation and ubiquitination in rose response to Botrytis infection
Source: Hortic Res. 2023 Nov 14;11(1):uhad238. doi: 10.1093/hr/uhad238 (PMC10782497; doi:10.1093/hr/uhad238)

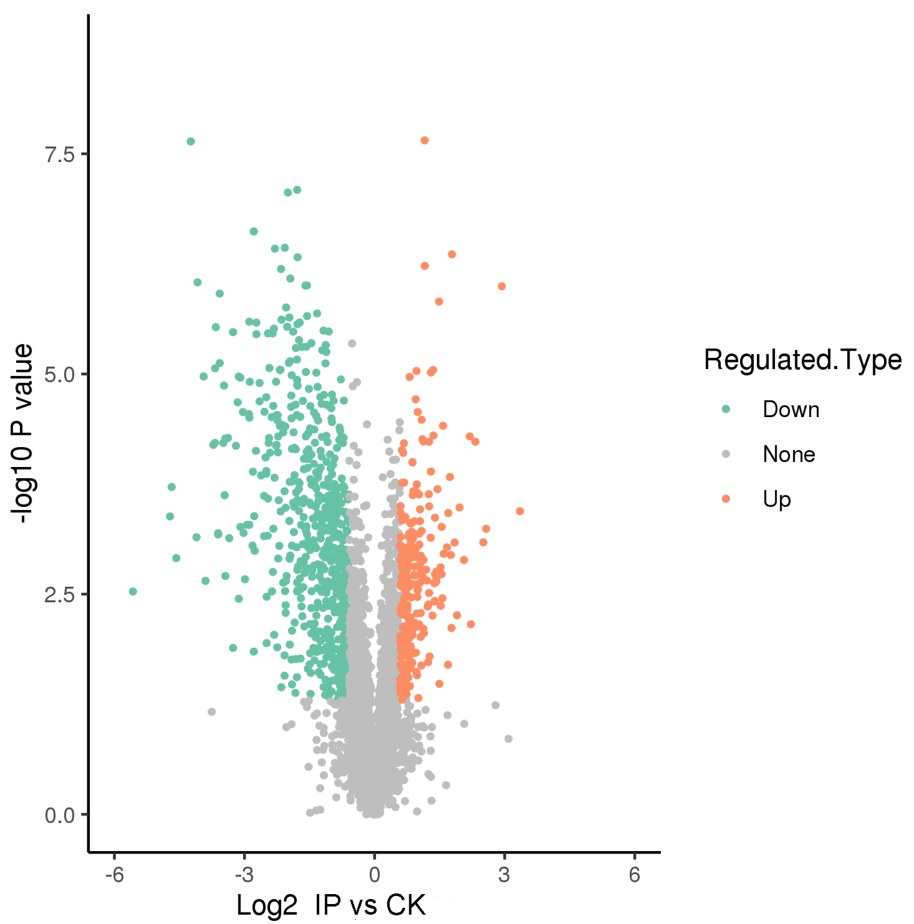


**Supplemental Figure S4** Volcanic map of differentially ubiquitinated sites.

Supplement: Web_Material_uhad238 [file web_material_uhad238.zip › Supplemental Figure S4.docx]
